# Destroying, vs. Capping the Nerve of Human Teeth

**Published:** 1848-04

**Authors:** B. T. Whitney


### DESTROYING, VS. CAPPING THE NERVE OF HUMAN TEETH.

BY DR. B. T. WHITNEY.

For more than nine years I have been in the habit of destroying the exposed nerves, usually with arsenic, then plugging the cavity with a view of saving, for a while at least, painful teeth. That practice, but a few years ago, was almost universally adopted; and still, almost universally followed; and found strenuous advocates, among the brightest ornaments of the profession; not simply for gain; but were happy to be able to relieve suffering humanity. It promised much, and the few teeth saved, have well compensated for the toil; but its indiscriminate use, in the hands of the empyric, for gain, reckless and ignorant of results, has produced an unnecessary amount of real suffering, and cast an opprobrium upon the profession. The application of arsenic, the most potent, or any other caustic for this object; it matters not what article or proportion of narcotic may be combined; is necessarily attended with a greater or less degree of irritation in the dental nerve, or peri dental membrane at the apex of the fang, that in a large majority of cases, at least, probably from six sevenths to nine tenths, will ultimately result in inflammation and suppuration, though they may do well for a short time.

The practice may have proved more successful in more skillful hands; though I am conscious of having treated them after the most approved plan for complicated caries.

Some years ago, I put in opposition to this practice, that of carefully placing a cap, sometimes of gold, sometimes of lead, over the exposed pulp, so as to prevent the pressure of the plug upon the sensitive nerve.

This I have found much more successful than destroying the nerve; having saved a majority of the teeth thus treated. But the indiscriminate application of this practice, to aching teeth, would be followed by a greater ratio of unfortunate cases. It is often extremely difficult to ascertain whether the nerve is in a healthy state, or from exposure and irritation, already an ichorous discharge is established. This being the case, it must have an outlet; and the unfortunate results are as well known to the intelligent operator before the experiment, as after subjecting the patient to expense and pain—namely, an alveolar abscess.

By the former practice, we are unable to destroy the nerve alone, but with it, an artery and vein, as necessary for the vitality of the tooth, as heat is to animal life: leaving the tooth simply a dead body, like any similar foreign substance, in the mouth. But by the latter, the entire support of the tooth is saved, the nerve is protected from external and injurious agents, and the functions go on, as in sound teeth.

Our systems of Physiology, speak strongly against such wanton interference in the ordinary functions of nature, and even a very limited knowledge of the anatomy and physiology of the human system, or of the denture alone, would carry with it a conviction at least, of the error of such a practice; and dental hygien has demonstrated it beyond a doubt.

The argument then is, that where actual disease in the pulp has not already interfered in the support of the tooth, we should not, by its total destruction; and only resort to that extreme, when disease is already established, and confined to the dental pulp, and then the "anchor of our hope" is, that the irritation produced by its destruction, may not carry the disease to the investing membrane; thus destroying what little support we had hoped to save the root.

The practice may be more justifiable, on the incisors and cuspidati, than on the bicuspides and molars, as I am led to believe that the investing membrane contributes more to the support of the former, than the latter; and the pain and irritation of killing the nerve is less severe; consequently, less danger of producing inflammation in the peridental membrane,—or *peridentis*, as it is proposed to call that disease.

I would like to hear the opinion of yourself, or of some of your able cotemporaries, on this subject.

*Clarksville, Tenn., Feb'y* 8, 1848.

We prefer always to save the nerve of a tooth, and at times have been very successful, by pursuing the course recommended by Dr. Koeker, as he remarks, "it requires much care in the removal of the caries, and indeed the whole treatment."

We consider the chance of success far more certain, when there has been no irritation of the nerve, and where, in removing the caries, the nerve is not wounded with the point of the excavating instrument.

Our practice is to cap the nerve with a piece of gold plate, formed so as to be a complete shield to the nerve, and then fill with gold—two metals are not admissible in the same tooth or cavity.

Under favorable circumstances like the above, no preparatory treatment is necessary before the operation of filling, except, indeed, washing of the cavity with tepid water, or tincture of Myrrh.

We apprehend the practice of killing the nerve with arsenic, and the like remedies, is nothing like so common as a few years since—under the best of treatment, many of such teeth must become painful ultimately. The most thorough treatment, and we think the best with this article is that of Dr. Maynard's, Washington City, and while with this we will succeed in many mouths, yet there are others, particularly of a nervous temperament, in whose mouths teeth thus treated will very soon become troublesome.

When perfectly practicable, the removal of the nerve with the cutting wire, presents to our view more hope of success than any other method. We know that front and bicuspid teeth can be cut off—the nerves thus destroyed, and if properly done, with judicious treatment thereafter, scarce ever inflammation supervenes. Just so have we found the removal of the nerve, when exposed in these teeth, and proper filings inserted.

This practice but few of our patients readily submit to, and yet, we think to preserve the color of the tooth, and prevent abscess, it is the most certain of any other.—CIN. ED.

